# A sampling-event dataset from the survey of wintering waterbirds in Coastal Fujian Province, China

**DOI:** 10.3897/BDJ.14.e197082

**Published:** 2026-07-30

**Authors:** Liangchen Ding, Jingpeng Cao, Lei Cao, Zheping Xu, Qingshan Zhao

**Affiliations:** 1 State Key Laboratory of Regional and Urban Ecology, Research Center for Eco-Environmental Sciences, Chinese Academy of Sciences, Beijing, China State Key Laboratory of Regional and Urban Ecology, Research Center for Eco-Environmental Sciences, Chinese Academy of Sciences Beijing China; 2 University of Chinese Academy of Sciences, Beijing, China University of Chinese Academy of Sciences Beijing China; 3 National Science Library, Chinese Academy of Sciences, Beijing, China National Science Library, Chinese Academy of Sciences Beijing China; 4 Department of Information Resources Management, School of Economics and Management, University of Chinese Academy of Sciences, Beijing, China Department of Information Resources Management, School of Economics and Management, University of Chinese Academy of Sciences Beijing China

**Keywords:** biodiversity monitoring, coastal wetlands, Darwin Core standard, sampling-event datasets, species distribution, waterbird conservation, waterbird population dynamics, winter census

## Abstract

**Background:**

Coastal wetlands in Fujian, eastern China, are an important component of the regional wetland system of south-eastern China and function as a key node along the East Asian-Australasian Flyway (EAAF). As a key segment of this migratory corridor, coastal wetlands in Fujian support substantial populations of migratory waterbirds by providing essential breeding habitats, stopover sites and stable overwintering refuges. Notably, coastal wetlands in Fujian support several IUCN threatened species, including the Endangered *Platalea minor* (Temminck & Schlegel, 1849) and *Anser cygnoides* (Linnaeus, 1758), as well as the Vulnerable *Aythya ferina* (Linnaeus, 1758) and *Chroicocephalus saundersi* Swinhoe, 1871. Therefore, maintaining the ecological integrity of coastal Fujian is critical for conserving biodiversity at regional and broader scales.

This dataset originates from a synchronised survey of wintering waterbirds conducted in coastal Fujian Province, China, in February 2006. The survey aimed to assess the distribution and abundance of waterbirds across the coastal wetlands of the region. The geographical extent of the survey ranged from 23°43′N to 27°17′N and from 117°19′E to 120°18′E, covering a total area of 2,657.56 km². Systematic observations were conducted at 191 fixed survey points within 21 wetlands, encompassing 25 wetland nature reserves. In total, 108,125 waterbirds were recorded during the survey, including 102,036 identified individuals belonging to 64 species and 39 genera. The top 10 species accounted for 84% of all individuals, comprising four shorebirds, three ducks, one gull, one egret and one cormorant. All records have been organised according to the Darwin Core standard, ensuring data comparability and reusability. This dataset provides essential information on the distribution patterns and abundance of wintering waterbirds in coastal Fujian, supporting long-term monitoring of species distributions and population dynamics, as well as waterbird conservation planning and policy-making.

**New information:**

Here, we present a comprehensive dataset on waterbirds from coastal wetlands in Fujian, China. The dataset comprises 64 waterbird species and 108,125 individuals recorded at 21 coastal wetland sites in February 2006. All observation records have been standardised following the Darwin Core format and the dataset has been made publicly available via the Global Biodiversity Information Facility.

## Introduction

Fujian Province lies on the south-eastern coast of China (23°33′N–28°20′N, 115°50′E–120°43′E). It forms an important section of the East Asian-Australasian Flyway and its distinctive geographical and ecological characteristics make it a key staging and wintering area for migratory waterbirds (Cheng et al. 2021, Huang et al. 2024). Fujian has a total coastline length of 6,128 km (including 3,324 km of mainland coastline and 2,804 km of island coastline), with over 90% of its provincial land area consisting of mountains and hills and 1,546 islands larger than 500 m², resulting in an extensive yet fragmented coastal morphology (Barter et al. 2007, Chen and Wen 2024, Huang et al. 2024). This coastal system not only provides important resting and breeding areas for waterbirds, but also contains scattered islands that serve as safe refuges from disturbance on the mainland.

Waterbirds are key bioindicators of wetland ecosystem health (Wang and Ma 2025). However, in recent decades, the numbers of migratory waterbirds using coastal wetlands in China have declined markedly (Wang et al. 2018, Ma et al. 2023). Coastal areas in Fujian Province have experienced extensive loss and degradation of wetlands and tidal flats driven by land reclamation, enclosure for cultivation, the expansion of aquaculture ponds and the historical invasion of *Spartina alterniflora* Loisel (Ma et al. 2014, Ma et al. 2019, Wu et al. 2022, Zheng et al. 2023). In estuaries and bays, roughly 75% of the total tidal-flat loss has occurred and the overall extent of tidal flats has decreased by about 20% (Wu et al. 2024). These changes have substantially reduced the capacity of coastal wetlands to support waterbirds and have posed serious risks to their survival, breeding and migration.

Although eastern China is an important wintering region for migratory waterbirds, the scarcity of both current and historical data on waterbird population sizes in this region severely limits our ability to assess the magnitude and drivers of their declines (Cao et al. 2008). Therefore, this study presents a survey dataset from the winter of 2006 in Fujian Province, documenting the distribution and abundance of wintering waterbirds in the province’s coastal areas. The data have been used in a range of published papers (Barter et al. 2007, Cao et al. 2008) and this dataset can serve as a reference for the development of a long-term standardised monitoring programme on population dynamics of coastal waterbirds in Fujian, while also providing a baseline for subsequent research and conservation planning.

## Sampling methods

### Study extent

The survey area encompassed the coastline of Fujian Province (23°43′N–27°17′N, 117°19′E–120°18′E). The 21 wetland survey sites varied widely in both spatial extent and habitat type, ranging from small sites of less than 10 km² (e.g. Yucheng Fish Ponds and Wenwusha Reservoir) to large estuarine bays exceeding 400 km² (e.g. Xinghua Bay and Sandu Bay). This sampling design maximises the capture of waterbird community patterns across a broad range of ecological settings and spatial scales.

### Sampling description

In this survey, the Waterbird Population Estimates 3^rd^ Edition (Delany and Scott 2002) and the Waterbird Population Estimates 5^th^ Edition (Mundkur and Nagy 2012) were used as the standard taxonomic references for the recognition of waterbird species. These documents serve as the standard references for confirming waterbird populations of international significance.

### Quality control

Existing research indicates that observers typically underestimate the numbers of large flocks of waterbirds (Rappoldt et al. 1985). Particularly across extensive wetland areas, undercounts resulting from visual obstruction and the birds' concealment behaviour remain a significant cause of bias in avian population estimates.

In this survey, large flocks of waterbirds were rarely observed, which helped improve the accuracy and reliability of species identification and population counts.

### Step description

Counts for this survey were carried out by two teams of experienced observers, each equipped with Zeiss 10 × 42 Victory roof prism binoculars and Leica Televid 77 telescopes with 20–60× zoom eyepieces.

Survey sites were selected using existing information on regional hydrology and waterbird habitats. Potential locations were initially identified using high-resolution satellite imagery and their coordinates were subsequently imported into GPS devices. Before the field survey, the investigators evaluated each potential site to determine the final survey locations and planned survey routes using GPS. Upon arrival at each target area, observation points were preferentially selected in leeward positions with wide, unobstructed views to maximise visibility. Observation distances were adjusted dynamically according to visibility; under favourable conditions, flocks within a radius of 4–5 km of each observation point could be counted. When visibility was poor, the density of observation points was increased to effectively reduce survey blind spots and minimise the risk of undercounting.

Survey teams departed for the field sites at approximately 08:00 h each morning, ensuring arrival at each wetland sampling point under favourable light conditions and during the morning peak of waterbird foraging activity, thereby facilitating accurate counts. Throughout the survey period, weather conditions were predominantly clear with good visibility, while wind speed and air temperature remained moderate, creating suitable conditions for waterbird counts.

The wetland sites included in the survey were readily accessible by road, allowing a comprehensive census to be completed within the designated survey period. Two main strategies were used to optimise coverage and efficiency: first, priority was given to vantage points that maximised the field of view; second, counts were concentrated during the high-tide period, when waterbirds congregate at high-tide roosts, to facilitate efficient enumeration.

## Geographic coverage

### Description

**Figure 1.**
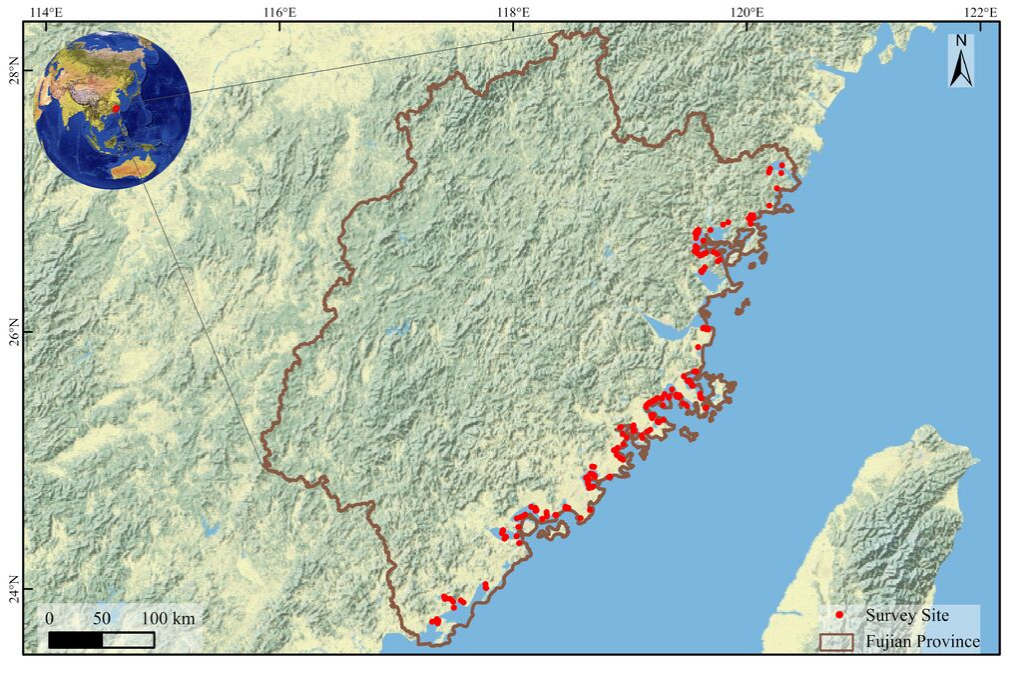
Geographic distribution of survey sites in coastal Fujian Province, China.

The present survey covered 21 coastal and offshore wetlands in Fujian Province, encompassing 25 wetland nature reserves, including four at the national level, four at the provincial level and the remainder at the prefectural or county level. In total, 191 survey sites were laid out within these wetlands (Fig. 1).

### Coordinates

23.7 and 27.3 Latitude; 117.3 and 120.3 Longitude.

## Taxonomic coverage

### Description

The survey was conducted over a 20-day period from 8 - 27 February 2006, covering 21 coastal and inshore wetlands in Fujian Province. A total of 108,125 waterbirds were recorded, including 6,089 unidentified individuals, with 64 species identified.

### Taxa included

**Table:** 

Rank	Scientific Name	
species	*Gavia stellata* (Pontoppidan, 1763)	
species	*Tachybaptus ruficollis* (Pallas, 1764)	
species	*Podiceps cristatus* (Linnaeus, 1758)	
species	*Podiceps nigricollis* C.L.Brehm, 1831	
species	*Pelecanus crispus* Bruch, 1832	
species	*Phalacrocorax carbo* (Linnaeus, 1758)	
species	*Ardea cinerea* Linnaeus, 1758	
species	*Ardea alba* Linnaeus, 1758	
species	*Bubulcus ibis* (Linnaeus, 1758)	
species	*Ardeola bacchus* (Bonaparte, 1855)	
species	*Egretta garzetta* (Linnaeus, 1766)	
species	*Nycticorax nycticorax* (Linnaeus, 1758)	
species	*Platalea leucorodia* Linnaeus, 1758	
species	*Platalea minor* Temminck & Schlegel, 1849	
species	*Cygnus columbianus* (Ord, 1815)	
species	*Anser cygnoides* (Linnaeus, 1758)	
species	*Tadorna tadorna* (Linnaeus, 1758)	
species	*Mareca penelope* (Linnaeus, 1758)	
species	*Mareca falcata* (Georgi, 1775)	
species	*Mareca strepera* (Linnaeus, 1758)	
species	*Anas crecca* Linnaeus, 1758	
species	*Anas platyrhynchos* Linnaeus, 1758	
species	*Anas poecilorhyncha* J.R.Forster, 1781	
species	*Anas acuta* Linnaeus, 1758	
species	*Spatula clypeata* (Linnaeus, 1758)	
species	*Aythya ferina* (Linnaeus, 1758)	
species	*Aythya fuligula* (Linnaeus, 1758)	
species	*Aythya marila* (Linnaeus, 1761)	
species	*Mergus serrator* Linnaeus, 1758	
species	*Mergus merganser* Linnaeus, 1758	
species	*Amaurornis phoenicurus* (Pennant, 1769)	
species	*Gallinula chloropus* (Linnaeus, 1758)	
species	*Fulica atra* Linnaeus, 1758	
species	*Haematopus ostralegus* Linnaeus, 1758	
species	*Recurvirostra avosetta* Linnaeus, 1758	
species	*Vanellus vanellus* (Linnaeus, 1758)	
species	*Pluvialis fulva* (J.F.Gmelin, 1789)	
species	*Pluvialis squatarola* (Linnaeus, 1758)	
species	*Charadrius dubius* Scopoli, 1786	
species	*Charadrius alexandrinus* Linnaeus, 1758	
species	*Charadrius mongolus* Pallas, 1776	
species	*Gallinago gallinago* (Linnaeus, 1758)	
species	*Limosa lapponica* (Linnaeus, 1758)	
species	*Numenius arquata* (Linnaeus, 1758)	
species	*Tringa erythropus* (Pallas, 1764)	
species	*Tringa totanus* (Linnaeus, 1758)	
species	*Tringa stagnatilis* (Bechstein, 1803)	
species	*Tringa nebularia* (Gunnerus, 1767)	
species	*Tringa ochropus* Linnaeus, 1758	
species	*Actitis hypoleucos* (Linnaeus, 1758)	
species	*Arenaria interpres* (Linnaeus, 1758)	
species	*Calidris alba* (Pallas, 1764)	
species	*Calidris ruficollis* (Pallas, 1776)	
species	*Calidris temminckii* (Leisler, 1812)	
species	*Calidris alpina* (Linnaeus, 1758)	
species	*Larus crassirostris* Vieillot, 1818	
species	*Larus hyperboreus* Gunnerus, 1767	
species	*Larus argentatus* Pontoppidan, 1763	
species	*Larus schistisagus* Stejneger, 1884	
species	*Chroicocephalus ridibundus* (Linnaeus, 1766)	
species	*Chroicocephalus saundersi* Swinhoe, 1871	
species	*Hydroprogne caspia* (Pallas, 1770)	
species	*Sterna hirundo* Linnaeus, 1758	
species	*Sternula albifrons* (Pallas, 1764)	

## Temporal coverage

**Data range:** 2006-2-08 – 2006-2-27.

## Usage licence

### Usage licence

Other

### IP rights notes

Creative Commons Attribution Non-Commercial (CC-BY-NC) 4.0 Licence

## Data resources

### Data package title

A sampling-event dataset from the survey of wintering waterbirds in Coastal Fujian Province, China

### Resource link

https://doi.org/10.15468/au2muq

### Alternative identifiers

https://www.gbifchina.org.cn/resource?r=birds_survey_2006

### Number of data sets

1

### Data set 1.

#### Data set name

A Sampling-event Dataset from the Survey of Wintering Waterbirds in Coastal Fujian Province, China

#### Data format

Darwin Core Event

#### Description

This dataset originates from the 2006 wintering waterbird survey conducted across 21 wetlands in the coastal regions of Fujian, China. It contains detailed species, abundance and geographical information (Ding et al. 2026).

**Data set 1.** 

Column label	Column description
eventID (Event Core)	An identifier for the broader dwc: Event that groups this and potentially other dwc: Events.
parentEventID (Event Core)	An identifier for the broader dwc: Event that groups this and potentially other dwc: Events.
samplingProtocol (Event Core)	The names of, references to or descriptions of the methods or protocols used during a dwc: Event.
sampleSizeValue (Event Core)	A numeric value for a measurement of the size (time duration, length, area or volume) of a sample in a sampling dwc: Event.
sampleSizeUnit (Event Core)	The unit of measurement of the size (time duration, length, area or volume) of a sample in a sampling dwc: Event.
samplingEffort (Event Core)	The amount of effort expended during a dwc: Event.
eventDate (Event Core)	The date-time or interval during which a dwc: Event occurred.
continent (Event Core)	The name of the continent in which the dcterms: Location occurs.
country (Event Core)	The name of the country or major administrative unit in which the dcterms: Location occurs.
countryCode (Event Core)	The standard code for the country in which the dcterms: Location occurs.
stateProvince (Event Core)	The name of the next smaller administrative region than country (state, province, canton, department, region etc.) in which the dcterms: Location occurs.
county (Event Core)	The full, unabbreviated name of the next smaller administrative region than stateProvince (county, shire, department etc.) in which the dcterms: Location occurs.
locality (Event Core)	The specific description of the place.
decimalLatitude (Event Core)	The geographic latitude (in decimal degrees, using the spatial reference system given in dwc: geodeticDatum) of the geographic centre of a dcterms: Location.
decimalLongitude (Event Core)	The geographic longitude (in decimal degrees, using the spatial reference system given in dwc: geodeticDatum) of the geographic centre of a dcterms: Location.
geodeticDatum (Event Core)	The ellipsoid, geodetic datum or spatial reference system (SRS) upon which the geographic coordinates given in dwc: decimalLatitude and dwc: decimalLongitude are based.
coordinateUncertaintyInMetres (Event Core)	The horizontal distance (in metres) from the given dwc: decimalLatitude and dwc: decimalLongitude describing the smallest circle containing the whole of the dcterms: Location.
eventRemarks (Event Core)	Comments or notes associated with each survey event, including counts of unidentified individuals that could not be assigned to species level, indications that no bird occurrences were recorded and notes on event-level data processing such as merged or re-assigned occurrence records.
basisOfRecord (Occurrence Extension)	The specific nature of the data record.
dynamicProperties (Occurrence Extension)	A JSON-formatted field used to record the IUCN Red List category of each taxon. The codes used in this dataset are LC = Least Concern, NT = Near Threatened, VU = Vulnerable and EN = Endangered.
occurrenceID (Occurrence Extension)	An identifier for the dwc: Occurrence (as opposed to a particular digital record of the dwc: Occurrence).
organismQuantity (Occurrence Extension)	A number or enumeration value for the quantity of dwc: Organisms.
organismQuantityType (Occurrence Extension)	The type of quantification system used for the quantity of dwc: Organisms.
occurrenceStatus (Occurrence Extension)	A statement about the presence or absence of a dwc: Taxon at a dcterms: Location.
scientificName (Occurrence Extension)	The full scientific name, with authorship and date information if known.
kingdom (Occurrence Extension)	The full scientific name of the kingdom in which the dwc: Taxon is classified.
phylum (Occurrence Extension)	The full scientific name of the phylum or division in which the dwc: Taxon is classified.
class (Occurrence Extension)	The full scientific name of the class in which the dwc: Taxon is classified.
order (Occurrence Extension)	The full scientific name of the order in which the dwc: Taxon is classified.
family (Occurrence Extension)	The full scientific name of the family in which the dwc: Taxon is classified.
genus (Occurrence Extension)	The full scientific name of the genus in which the dwc: Taxon is classified.
taxonRank (Occurrence Extension)	The taxonomic rank of the most specific name in the dwc: scientificName.
